# A case of aggressive giant dermatofibrosarcoma protuberance occurring in the parotid gland

**DOI:** 10.1016/j.ijscr.2018.12.006

**Published:** 2019-01-22

**Authors:** Dauren Adilbay, Fariza Khozhamkul, Samal Toiynbekova, Daniar Ahmetov

**Affiliations:** Head and Neck Oncology Center, Kazakh Institute of Oncology and Radiology, 91 Abay Ave, Almaty, Kazakhstan

**Keywords:** Dermatofibrosarcoma protuberans (DFSP), Parotid gland, CD34, ALT flap, Case report

## Abstract

**Introduction:**

Dermatofibrosarcoma protuberans (DFSP) is a cutaneous malignancy that arises from the dermis and invades deeper tissue. The cellular origin of DFSP is not clear. Evidence supports the cellular origin being fibroblastic, histiocytic, or neuroectodermal.

**Presentation of case:**

A 38 years old, women presented with slow-growing large right parotid mass. A total parotidectomy performed with auriculectomy and reconstruction using ALT flap. Diagnosis confirmed by pathology and immunohistochemistry. Tumor recurred in 10 months, the second surgery with subsequent chemoradiotherapy performed. Patient initially treated with wide resection, 4 cycles of chemotherapy and postoperative radiotherapy 40 Gy, with the recurrence in 10 months. We performed a second surgery followed by radiotherapy. She is disease-free for more than two years under follow-up.

**Discussion:**

The main treatment of DFSP is surgical resection with wide negative margins or Moh’s surgery. Advanced cases treated with addition of radiotherapy or chemoradiotherapy, but with unclear benefits. In our case, huge tumor located in the parotid region recurred after initial surgery and adjuvant treatment.

**Conclusion:**

Clinically, DFSP usually manifest as well circumscribed, slow-growing, smooth, and painless masses. In cases with advanced tumor in parotid region, recurrence may occur despite aggressive initial treatment with wide resection and chemoradiotherapy.

## Highlights

• The parotid region is a very rare site with few published case reports, shares the common features of trunk DFSP.
• Radiotherapy or chemoradiotherapy can be applied to large and recurrent cases, but with unclear benefit.
• Dermatofibrosarcoma protuberans is a rare tumor, with infiltrative margins, high local recurrence rate, and rare distant metastasis.
• CD34 immunohistochemistry is a reliable marker for difficult diagnostic cases.

## 1 Introduction

Although DFSP may have been reported in the literature as early as 1890, Darier and Ferrand first described it in 1924 as a distinct cutaneous disease entity called progressive and recurring dermatofibroma. Hoffman officially coined the term dermatofibrosarcoma protuberans in 1925 [1]. Dermatofibrosarcoma protuberans (DFSP) is a relatively uncommon soft tissue neoplasm of intermediate- to low-grade malignancy. The incidence of sarcomas of the major salivary gland appears to be about one-tenth of benign mesenchymal tumors [2,3]. Metastasis occurs rarely. DFSP is a locally aggressive tumor with a high recurrence rate [1]. The treatment of choice is surgical resection with wide margins or Mohs micrographic surgery [4,5]. In cases of large, unresectable, metastatic tumors, the adjuvant radiotherapy or chemotherapy implemented as described in several papers [6]. Tyrosine-kinase inhibitors like imatinib or sorafenib use are more efficient compared with other classical agents [6]. We report a very rare case of large DFSP located in the parotid region with involvement of the parotid salivary gland, external ear requiring large resection volume and immediate reconstruction with free ALT flap.

The work has been reported in line with the SCARE criteria [7].

## 2 Presentation of case

**Fig. 1:**
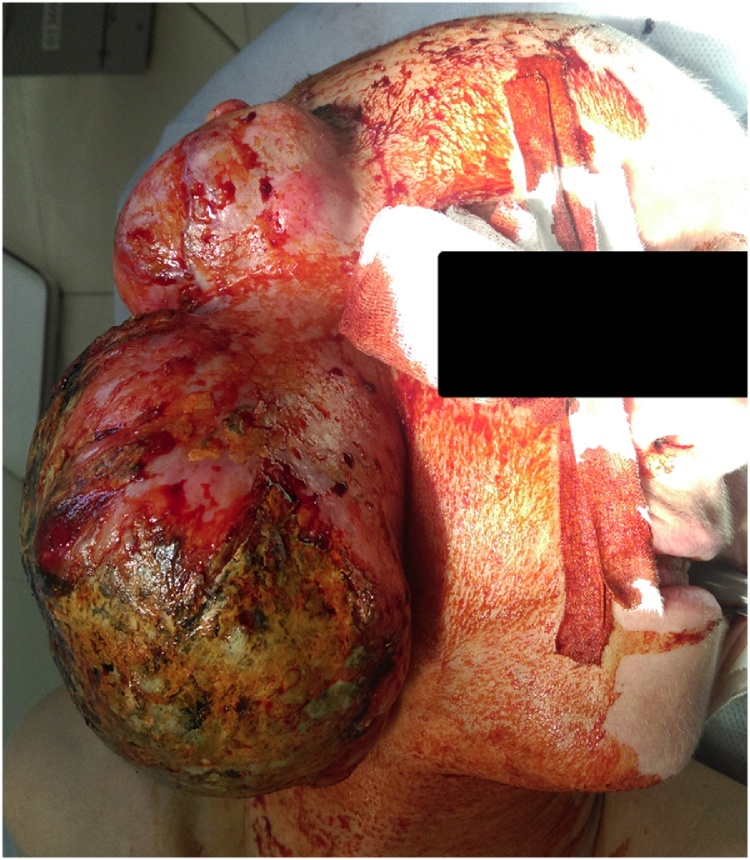
Patient appearance with right parotid region tumor.

A 38-year old Russian woman presented with the complaint of a slow growing, painless pretragal swelling of eight years duration. The patient had a resection of parotid gland under local anesthesia eight years ago in the local regional hospital. Microscopic examination showed signs of angiofibrosis. In 1 year there was a recurrence of the tumor, and histological examination did not reveal any malignant cells. After seven years the patient noticed the fast growth of the tumor. General practitioner referred the patient to our institute, tertiary research cancer center. The clinical examination found a dense, slightly painful on palpation mass in the right parotid region with exophytic growth on a wide base with spread to the temporal, mastoid region and extension to the auricle cartilage measuring 18.0 × 8.0 × 9.0 cm. There was no facial paralysis or cervical lymph node enlargement (Fig. 1).

**Fig. 2:**
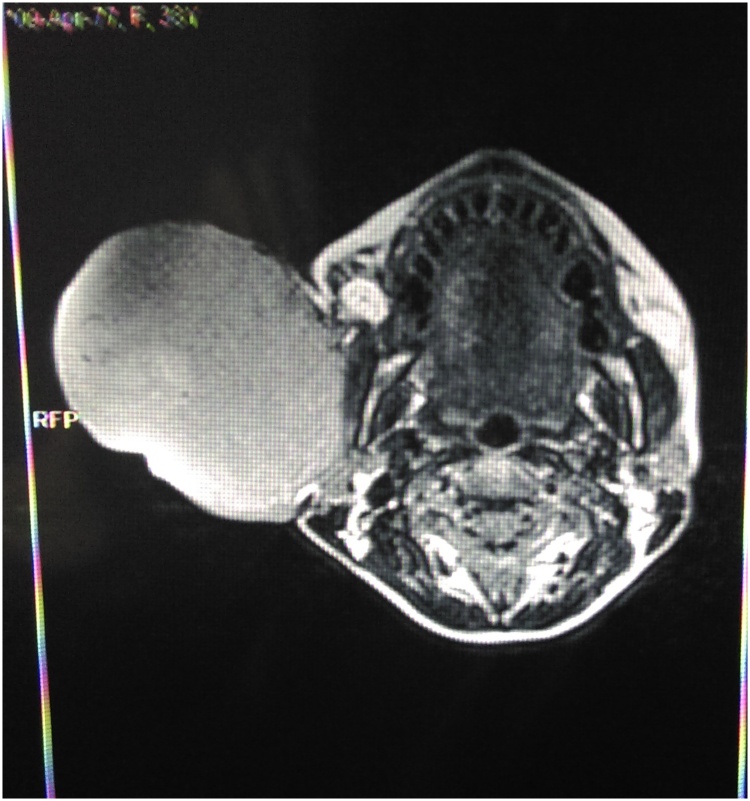
MRI scans showing a right parotid mass.

MRI scan revealed the homogeneous mass in the right parotid region with good margins, unmineralized, nodular soft-tissue mass involving the skin and subcutaneous adipose tissue. The features were suggestive of a sarcoma (Fig. 2).

According to, the decision of the tumor board, wide resection of the tumor including right total parotidectomy, auriculectomy, and the reconstruction of the postoperative defect with the anterolateral thigh flap on the microvascular anastomosis performed.

**Fig. 3:**
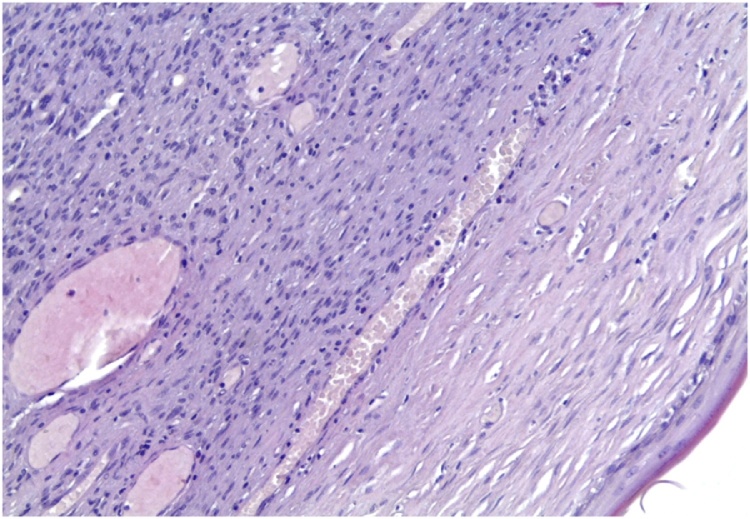
The microscopic appearance of the tumor.

**Fig. 4:**
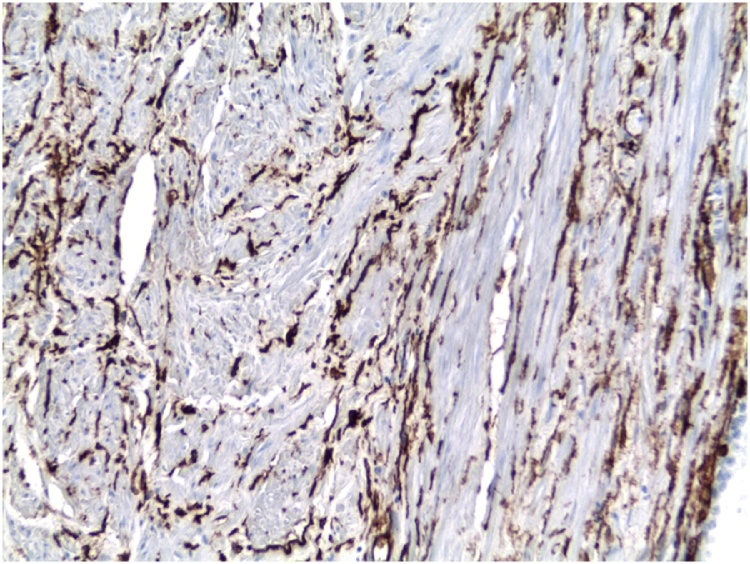
CD34 immunohistochemistry of the tumor x200.

Histopathological examination revealed short spindle cells, hypercellularity, moderate to marked atypia, nuclear pleomorphism and high mitotic activity with the tight storiform pattern (Fig. 3). On immunohistochemistry tumor, diffusely CD34 positive (Fig. 4). The final histopathological diagnosis confirmed as dermatofibrosarcoma protuberance.

In postoperative period she decided to get treatment in a regional cancer hospital. Four cycles of dacarbazine 400 mg on 1–5 days, doxorubicin 100 mg and 40 Gy radiotherapy delivered to the primary tumor bed by her regional cancer hospital. Tumor recurred after ten-month follow-up. The PET-CT examination found a 4 cm mass in the right parotid area.

**Fig. 5:**
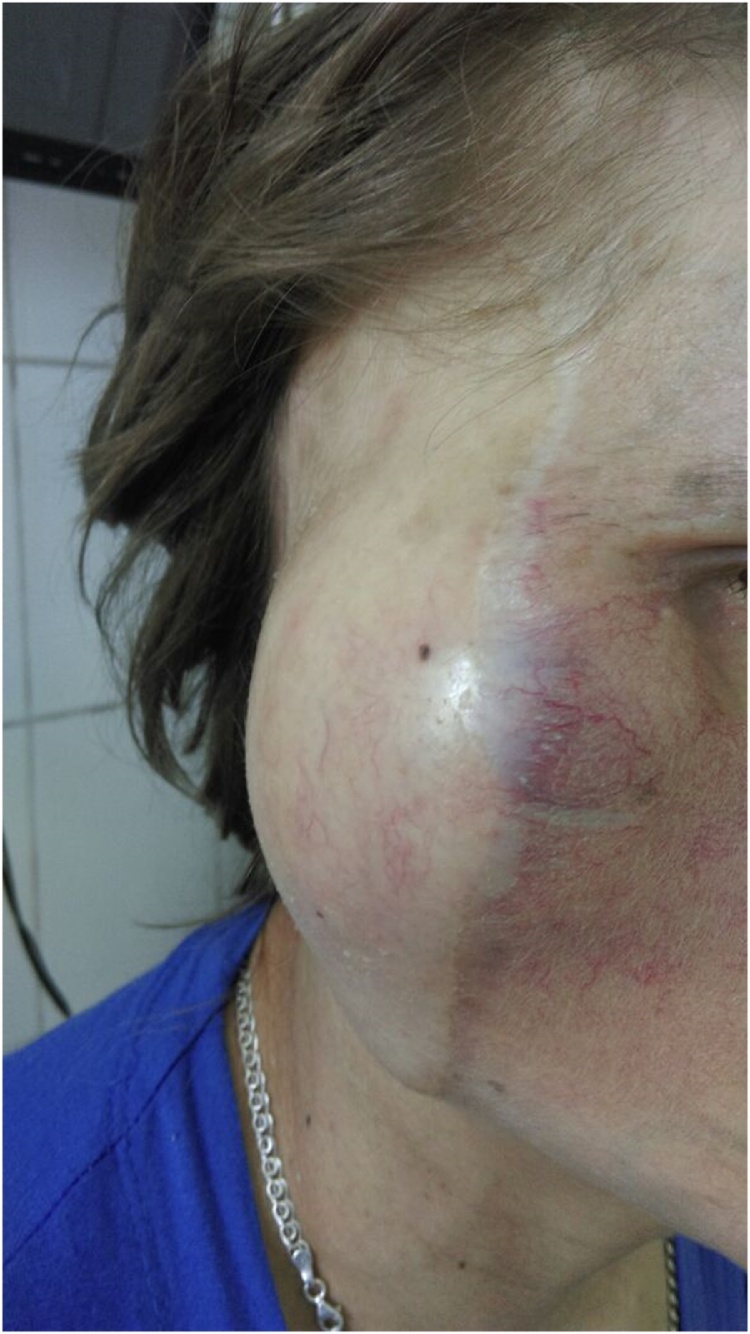
Patient appearance with recurrence, ten months after surgery.

Resection of recurrent tumor performed without complications (Fig. 5). The microscopic picture showed signs of dermatofibrosarcoma. The adjuvant radiotherapy till 80 Gy performed after the second resection. TKIs as Imatinib or Sorafenib not used, as was unavailable in a regional cancer hospital. She refused to continue treatment in Almaty due to her own reasons. The patient is under follow-up and disease-free for 28 months after the last treatment.

## 3 Discussion

DFSP is a fibrohistiocytic tumor of low to intermediate malignancy, having infiltrative margins with high local recurrence (about 50%), and rare distant metastasis [8]. These tumors occur mainly over the trunk and proximal extremities and tend to recur after wide local excision. Parotid gland or parotid region is a very rare site with few published case reports [2].

Routine workup consists of MRI (preferable) or CT and biopsy with histological examination. On computed tomography, these tumors appear as well-defined masses that are hypointense to muscle and demonstrate homogenous contrast enhancement. On magnetic resonance imaging, DFSP is homogeneous and iso- or hypo-intense to muscle. They are strongly enhanced post-contrast on T2-weighted images [9].

The macroscopic characteristics of the tumor include a well-defined and encapsulated mass which may be accompanied by bone destruction, though it is normally free of infiltration.

Histological features suggesting malignancy include high mitotic rate (four or more mitoses in 10 high power fields), hypercellularity, moderate to marked atypia and nuclear pleomorphism, tumor necrosis and infiltrative borders. Histologically, a diagnosis of DFSP is also difficult because many tumors display similar findings. DFSP, unlike a solitary fibrous tumor, shows remarkable uniformity, a lack of the hemangiopericytic pattern and a distinct storiform pattern around an inconspicuous vasculature. Because of overlapping immunohistochemical results for DFSP and solitary fibrous tumor, caution required in the differential diagnosis. Benign fibrous histiocytoma may resemble DFSP, but it is usually negative for CD34 and Bcl-2. Schwannoma contains Antoni A and B areas, and it is S-100 protein positive which not seen in DFSP [10]. Molecularly, DFSP characterized by a specific t(17;22)(q22;q13) translocation leading to the formation of COL1A1-PDGFB fusion transcripts [10].

The main treatment of DFSP is surgical resection with wide negative margins. Due to the giant size of the tumor in our case, we decided to carry out the one-stage reconstruction of the postoperative defect with the anterolateral thigh flap on the microvascular anastomosis. Mohs technic recommended by several publications gives the possibility to have narrow margins [11].

The 5-year survival rate for head and neck sarcomas is approximately 50%. Most authors agree that the same prognostic factors– grade, size, and depth-apply to sarcomas no matter where they arise. In the head and neck, however, local recurrence has more significant consequences because of the difficulty of subsequent management. In general, salivary gland sarcomas are aggressive neoplasms with recurrences in about 40–64%, hematogenous metastasis and mortality rates ranging from 36 to 64%. The prognosis for parotid DFSP is not clear due to the scarcity of cases reported in the literature [2].

In our case recurrence was observed within 11 months after treatment despite aggressive postoperative treatment including 4 cycles of chemotherapy and 40 Gy radiotherapy. After second surgery only radiotherapy performed as adjuvant therapy [12]. Some studies also recommend adjuvant tyrosine-kinase inhibitors therapy with good response rates published by several authors [5,6]. Only both surgeries performed by our team, all postoperative treatment delivered by regional cancer hospital without access to TKIs.

Since recurrence and metastasis can take place after several years, a lifelong clinical and imaging regular follow-up is compulsory.

## 4 Conclusion

Dermatofibrosarcoma protuberans is a rare tumor, with infiltrative margins, high local recurrence rate, and rare distant metastasis. Parotid gland or parotid region is a very rare site with few published case reports, shares the common features of trunk and extremities DFSP. The complete resection is the most important prognostic factor, and no evidence supports the efficacy of any therapy different to surgery. Radiotherapy or chemoradiotherapy can be applied to large and recurrent cases, but with unclear benefit. Due to the frequent local recurrence even after many years of remission, a long-term follow-up is guaranteed.

## Conflicts of interest

No conflicts of interest to declare.

## Sources of funding

It is a case report study. No funding was obtained to perform a study.

## Ethical approval

Not applicable, observational case reports are exempt from ethical approval in our Institution.

## Consent

We have obtained a written consent from a patient; despite there is no possibility to identify a patient on provided pictures.

## Author contribution

DA – study concept, and final approval. DA, FK, ST – acquired and interpreted the data and drafted the manuscript. DA, DAh. - performed the operation and perioperative management of the patient, revision of the manuscript. All authors read and approved the final manuscript.

## Registration of research studies

Not applicable.

## Guarantor

Dauren Adilbay.

## Provenance and peer review

Not commissioned, externally peer-reviewed.
